# Effects of Probiotic Supplementation on Sports Performance and Performance-Related Features in Athletes: A Systematic Review

**DOI:** 10.3390/ijerph20032226

**Published:** 2023-01-26

**Authors:** Mirella Di Dio, Patrizia Calella, Concetta Paola Pelullo, Fabrizio Liguori, Valeria Di Onofrio, Francesca Gallè, Giorgio Liguori

**Affiliations:** 1 Department of Movement Sciences and Wellbeing, University of Naples “Parthenope”, 80133 Naples, Italy; 2 Department of Economics and Legal Studies, University of Naples “Parthenope”, Via Generale Parisi 13, 80132 Naples, Italy; 3 Department of Sciences and Technologies, University of Naples “Parthenope”, 80143 Naples, Italy

**Keywords:** athletes, sport, performance, probiotic, diet supplementation

## Abstract

This review aims to evaluate the effects of probiotic supplementation on performance and performance-related conditions in athletes by evaluating randomized controlled studies from the MEDLINE (Pubmed), Web of Science, Scopus, and SPORTDiscus (EBSCO) databases. From a total of 2304 relevant articles, 13 studies fulfilled the inclusion criteria. Seven studies concern endurance athletes, one to rugby players, three refer to non-specified athletes, one to badminton players, and one involves baseball players. The evidence suggests that the integration of athletes’ diets with some bacterial strains and also the consumption of multi-strain compounds may lead to an improvement in performance and can positively affect performance-related aspects such as fatigue, muscle pain, body composition, and cardiorespiratory fitness. However, the type of supplementation and sport is very variable among the studies examined. Therefore, to obtain more solid evidence, further controlled and comparable studies are needed to expand the research regarding the possible repercussions of probiotics use on athletes’ performance.

## 1. Introduction

It is known that physical activity (PA), which includes any form of movement in which the contraction of skeletal muscles results in an increase in energy consumption, has numerous beneficial effects on human health [1]. Regularly performing endurance and muscle-strengthening PA can improve physiological parameters which in turn lead to an enhancement in health-related physical fitness, a physiologic state of well-being related to health status including cardiovascular fitness, musculoskeletal fitness, body composition, and metabolism [1,2]. PA is also able to maintain or improve human neurocognitive conditions, and strengthen immune defences [2]. Scientific evidence has underlined the preventive role of regular physical activity towards chronic diseases such as diabetes, cancer, and cardiovascular diseases and related premature death [1,2].

Physical exercise is aimed to improve body functionality through adaptation. Current evidence suggests that physical exercise can affect, in quantitative and qualitative terms, the intestinal microbiota composition. It seems, in fact, that physical exercise generates greater microbial diversity in the gut, increases the Bacteroidetes-Firmicutes ratio, stimulates the proliferation of bacteria that can modulate mucosal immunity, and improves the intestinal barrier functions, with beneficial effects on the health of the host [3,4,5,6,7,8,9,10,11,12].

However, it has been observed that high-intensity exercise, which is common in individuals practising sports, can have detrimental effects on health. It can cause an increase in intestinal permeability and a decrease in the thickness of the intestinal mucus, potentially allowing pathogens/toxins to enter the bloodstream; moreover, it has been associated with immunosuppression by decreasing the function of immune cells, which improves susceptibility to infections [13,14,15,16,17,18,19], such as upper respiratory tract infection (URTI) [19,20,21]. This can be related to acute immune failure and chronic suppression of immune factors which follow frequent and strenuous exercise [22,23].

In addition, during intense training and competitions, gastrointestinal (GI) disorders, such as diarrhoea and heartburn [17,18], can occur. Causing interruptions in training or competitions, the aforementioned pathologies can have a negative indirect impact also on athletic performance [24,25]. Therefore, the reduction of these effects on athletes becomes a top priority.

In recent years, there are growing studies supporting the efficacy of taking probiotics, microorganisms which can have beneficial effects on the organism, in reducing the incidence and severity of acute infectious diarrhoea and Upper Respiratory Tract Infections (URTIs) in the general population [26,27,28]. These effects derive from the modulation of the immune system operated by probiotics. Furthermore, probiotics are also modulators of the intestinal microbiota, which can also have an indirect influence on various indices of physical performance and subsequent recovery. A recent systematic review has demonstrated the beneficial effects of probiotics in reducing the risk of developing GI and respiratory infectious diseases or the severity of symptoms associated with these disorders also in athletes [29]. Therefore, supplementation with probiotics could have positive effects on athletes’ health [25,30].

Besides the evidence regarding the effects on the athletes’ immune defences, it is not clear, however, if and which type of probiotic supplementation may lead to beneficial effects on sports performance in athletes. In particular, its possible effects on all the major dimensions of sports performance (skill, strength, endurance, and recovery) should be investigated from this point of view. Furthermore, since physical fitness provides the basis for sports performance [1], it is interesting to assess which performance-related physiological parameters can be affected by probiotic supplementation in athletes.

To address these questions, this review aimed to critically analyze the literature regarding the effects of probiotics supplementation on performance and performance-related conditions and characteristics in athletes.

## 2. Materials and Methods

The systematic review was performed according to the Preferred Reporting Items for Systematic Reviews and Meta-Analyses (PRISMA) guidelines [31]. The Review protocol was registered on PROSPERO (CRD42021268105).

### 2.1. Eligibility Criteria

The selection of the studies was performed through the PICO model, by considering the following parameters: P (patients) healthy adult athletes; I (intervention) diet supplementation with probiotics; C (comparison) comparison with a control group; O (outcomes) potential effects of probiotic supplementation on performance. Only randomized controlled studies performed on healthy adult athletes with a description of probiotics supplementation were included. The inclusion and exclusion criteria are shown in Table 1.

### 2.2. Literature Search and Selection of Studies

The article search was carried out by keywords (probiotic AND sport OR exercise OR athletes OR physical activity). Further articles were also searched in the reference lists of available reviews. Articles in English, Spanish, Italian and French languages were considered. The search was completed at the end of July 2022.

### 2.3. Data Collection

The articles found by database search were independently assessed by two reviewers (M.D.D and P.C.). Duplicate articles across the different databases were excluded, and then potentially eligible studies were identified by title and abstract screening. The same reviewers analyzed independently the full texts of these articles and selected those studies that met the selection criteria. Another reviewer (V.D.O.) resolved the disagreements.

Two other reviewers (G.C. and C.P.P.) extracted the following information from each selected study: authors and year of publication, study design, sample size and demographic characteristics of participants, characteristics of probiotic supplementation, outcomes and main results concerning sports performance and physical conditions or characteristics related to performance.

### 2.4. Risk of Bias

The revised Cochrane Risk-of-Bias tool for randomized trials (RoB2) was used to assess the risk of bias in the selected studies [32]. The evaluation was performed by two reviewers (M.D.D. and P.C.). Conflicts were resolved by a third researcher (F.G.). The risk of bias for each study was defined as low, moderate, or high.

## 3. Results

### 3.1. Article Selection and Characteristics

**Figure 1:**
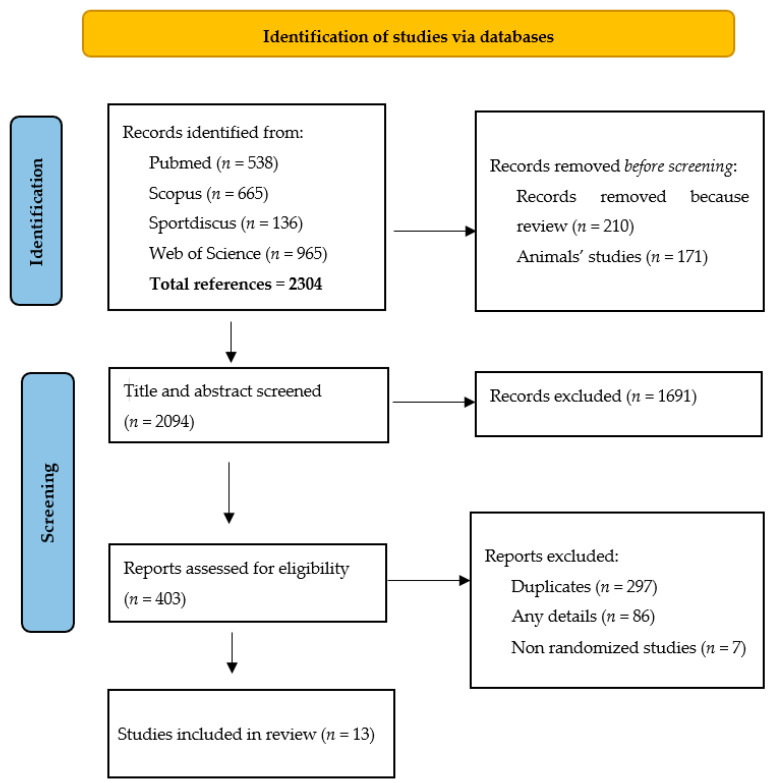
Prisma flow diagram of the article selection.

A total of 2304 relevant articles were initially found (Figure 1). Of these, 403 were considered eligible. After eliminating duplicates from the different databases and non-randomized studies, 13 articles [33,34,35,36,37,38,39,40,41,42,43,44,45] were selected considering the inclusion and exclusion criteria (Table 1).

The selected studies were performed between 2014 and 2021 in different geographic areas: Europe (Austria, UK, Spain, Poland), West Asia (Israel), East Asia (Malaysia, Japan, Taiwan), North America (USA, Tennessee) and Oceania (Australia). The majority of them had a double-blind, placebo-controlled design.

Table 2 shows the characteristics of each study. Three studies did not report the participants’ gender [34,38,43], eight studies involved only males [33,35,36,37,39,40,41,45], one study involved only females [44] and one study included both genders [42]. The mean age was included between 19.5 ± 1.0 and 37.21 ± 8.9 years.

Seven studies concern endurance athletes [34,35,37,39,40,41,42], one study concerns rugby players [33], three studies refer to non-specified categories of athletes [36,43,44], one study concerns badminton players [38] and one study involved subjects who played baseball [45]. In seven studies [34,35,36,38,39,44,45] the probiotic supplementation was represented by a single bacterial strain, while in the remaining studies, it included the consumption of a multi-strain compound.

Regarding the risk of bias assessment, eight out of thirteen studies showed a low risk of bias (Table 3).

### 3.2. Outcome

Effects of Probiotic Supplementation by Type of Sport

Studies on Endurance Athletes

The effect of probiotics supplementation on endurance athletes’ performance was investigated in seven studies.

In two studies by Huang et al. improvements in running performance, due to a modulation of the microbiota and related metabolites [35], and better training management were registered after the integration of a single bacterial strain probiotic for 3–4 weeks in triathletes [34]. Shing et al. have found that an integration of a bacterial multistrain probiotic, for 4 weeks in male runners can lead to an extension of the performance execution times before reaching a condition of fatigue [41]. In the other two studies, however, supplementation with probiotics seems to have had no effect on performance. In both studies, supplementation involved the administration of a bacterial multi-strain probiotic for a period of 28 days [37] in one and 90 days in the other [40].

Smarkusz-Zarzeka et al., on the other hand, also investigated the effects of supplementation of a multi-strain for a period of 3 months. They investigated maximum oxygen consumption, for which there was an increase in both males and females [42]. The latter result is in disagreement with the study conducted by Huang et al., in which the integration of a probiotic with a single bacterial strain, for a period of 4 weeks, did not show an increase in maximum oxygen consumption [35].

Sawada et al. concluded that supplementing a single-strain probiotic for a period of 12 weeks in male runners is effective in recovering from fatigue but does not cause any significant difference in physical performance [39].

Studies on rugby athletes

Harnett et al. conducted the experimentation on male athletes and deduced that the supplementation administered (multispecies probiotic for 17 weeks) led to a reduction of muscle pain and heaviness in the legs and all these correlate to an improvement in the quantity/quality of sleep and motivation during training and competitions [33].

Studies on badminton athletes

Salleh et al. conducted the trial on a group of players for which no gender was specified, who were given a single strain probiotic for a period of 6 weeks. Supplementing probiotics improved aerobic capacity but did not affect speed, strength, leg power and agility. Furthermore, after six weeks, the anxiety and stress levels of athletes significantly decreased by 16% and 20%, respectively, compared to pre-intervention conditions [38].

Studies on baseball athletes

Towsend et al. examined the effects of 12 weeks of daily single-strain probiotic supplementation on male athletes’ immune and hormonal profiles and physical and performance adaptations during a period of increased academic and physical stress. Results showed that the supplementation had no effect on body composition, performance, hormonal status, or intestinal permeability, but it produced a significant decrease in circulating TNF-alpha levels, which could indicate an anti-inflammatory action of the probiotics [45].

Studies on Athletes Practicing Other Types of Sports

Komano et al. conducted a trial on a group of male athletes who were given a single-strain probiotic for a period of 13 days. In accordance with the hypotheses formulated by the authors, the results indicated that this integration would be able to increase the marker of dendritic cell maturation (CD86) and could reduce cumulative days of fatigue [36]. Strasser et al. investigated the effects of a multi-strain probiotic supplementation for 3 months on a group of athletes, for which no gender was specified, and found no effect on physical performance [43]. Toohey et al. instead administered a single-strain probiotic for 10 weeks to female athletes. In this case, the supplementation had no direct effect on performance, but it improved the body composition of the athletes [44].

## 4. Discussion

This review analyzed interventions conducted from 2014 to 2021, which highlights the recent interest towards probiotic supplementation in sports. These studies look at the effects of probiotics on different performance features, in different sports disciplines. Studies on endurance sports and on male athletes were the most represented.

Overall, 11 out of 13 studies reported positive, although not always statistically significant, effects of probiotic supplementation on different aspects of sports performance.

Considering the interventions based on the use of single-strain products, it seems that probiotic supplementation induced positive actions in terms of endurance, aerobic capacity and fatigue recovery.

In particular, for the *Lactobacillus genus*, the integration of *Lactobacillus shirota* resulted in better aerobic capacity in badminton players [38]; the integration of *Lactobacillus plantarum* produced an ergogenic effect in triathletes, better maintenance of physical performance and endurance running performance [34,35]; *Lactobacillus gasseri* supplementation in runners was effective in recovering from fatigue but did not cause any significant differences in physical performance [39].

As for the integration of probiotics based on *Bacillus subtilis*, in volleyball/soccer players its integration has favoured an improvement in physical composition but not in performance, expressed as strength and agility [44]; while its integration using a lower dose in male baseball players did not produce any improvement in terms of physical composition and performance [45]. This could suggest that the effects of this strain could be dose-dependent.

Regarding multispecies probiotic supplementation, it was used in six of the studies included in this review [33,37,40,41,42,43]. Even in the majority of these studies, the results of supplementation were encouraging [33,40,41,42]. In particular, the consumption of a probiotic based on strains belonging to the genera *Lactobacillus*, *Bifidobacterium* and *Streptococcus* for a period of 17 weeks, at a dose of 60 billion, resulted in a reduction of muscle pain and heaviness of the legs in rugby players and consequently there was also an improvement in motivation during training and competitions. Also in male runners, who were given a probiotic based on strains belonging to *Lactobacillus*, *Bifidobacterium* and *Streptococcus*, albeit for a shorter period of time and at a lower concentration, there was an increase in the execution time of physical exercise, before reaching a condition of fatigue. Positive results were also obtained following the integration, in a group of runners, with a probiotic compound based on strains belonging to the genera *Lactobacillus*, *Bifidobacterium* and *Lactococcus*. In particular, there was an increase in O2 consumption (VO2 max), minute ventilation, exercise capacity, respiratory reserve and functional capacity in men. In the study by Schreiber et al., the administration of a multistrain compound including the genera *Lactobacillus*, *Bifidobacterium*, *Enterococcus* and *Bacillus* to male cyclists led to a reduced rate of perceived exertion. In all these studies, the probiotic administered showed a great heterogeneity of strains, suggesting that this could have higher beneficial effects than supplements containing single or similar strains.

The main limitations of this study are related to the heterogeneity of the selected articles. In fact, probiotics were administered in different quantities and forms and the length of the intervention was also different in the studies examined, sometimes performed during training and sometimes during competition phases. Furthermore, as far as the samples examined in the studies are concerned, the male gender is far more represented than the female one, and almost all the interventions interested young adults. Exploring the possible effects of probiotics on the sports performance of females and other age groups is essential to increase knowledge in this field.

The strengths, however, are related to the quality of the evidence, since only randomized studies were included, most of which showed a low risk of bias.

Unfortunately, the number of studies available in this area is still small. In fact, the research has directed its interest more towards the use of probiotics on gut health and has underestimated the potential effects that they could have on the different domains of performance. With regard to this, it should be considered that this systematic review was aimed at exploring a relatively recent research field, taking into account all the possible aspects related to performance in different sports. As the volume of the literature on this subject grows, it will be possible in the future to carry out more detailed analyses on specific performance domains and on specific athletes’ categories. Further research should also allow us to study in depth the precise role of each specific bacterial strain assumed alone or in combination with others, to establish which can be the most effective probiotic formulation to achieve specific results on the basis of sport and athletes’ characteristics.

Finally, given the mostly positive results found so far, it might be useful to further investigate the possible increase in physical performance determined by the use of probiotics, in the context of possible doping effects of probiotics [46,47].

## 5. Conclusions

In conclusion, the evidence coming from the studies analyzed suggests that the integration of athletes’ diets with some bacterial strains and also the consumption of multi-strain probiotic compounds may lead to an improvement in some performance domains, such as endurance, strength and recovery, and in performance-related physical conditions and characteristics, such as muscle pain and body composition. However, the heterogeneity of the available studies does not allow us to draw definite conclusions on this issue. Interest in this field of exploration is growing but the available studies cannot still be compared to provide solid evidence. To verify the effects of probiotic supplementation on sports performance, further controlled and comparative studies are needed.

## Figures and Tables

**Table 1:** Inclusion and exclusion criteria used in the article selection.

Inclusion Criteria	Exclusion Criteria
Randomized controlled studies Studies on healthy adult athletes Studies that evaluated probiotics’ effects on performance and/or on performance-related physiological parameters Studies based on probiotic supplementation as an intervention Studies reporting the type and dose of probiotic supplementation	Studies on animals Studies on non-healthy individuals Studies on non-physically active individuals Studies performed on children Studies not reporting supplementation details

**Table 2:** Characteristics of the included studies.

Author, Year, Country, Study Design	Sample Characteristics N of Subjects_ M/F_ Mean Age	Probiotics Daily Intake Intervention Length	Type of Performance/Physical Condition Variables Studied	Main Results
Harnett, 2020 Australia -double-blind randomised controlled trial	Elite male rugby union players Probiotic group (*n* = 9) 27.0 ± 3.2 years Placebo group (*n* = 10) 26.6 ± 2.9 years	genera *Lactobacillus, Bifiodbacterium* and *Streptococcus* (during international competition) Dose: 60 bilion SBFloractivTM (Bioceuticals, Australia AustL# 285024) containing 250 mg of the yeast *Saccharomyces boulardii* was added to the probiotic regime during the international travel −17 weeks	Muscle soreness rated on a 1–5 scale: 5 = non-existent, 4 = fine, no dramas let’s get on with it, 3 = there’s some, but after normal warm-up, I’ll be fine, 2 = pretty ordinary, my movements are stiff and sore, and1 = severe, I need to alert the physio or doctor. Leg heaviness on a 1–10 Likert scale	Muscle soreness was ∼0.5 units lower (F(1, 343) = 42.646, *p* < 0.0001) and leg heaviness scores∼0.7 units lower (F(1, 334) = 28.990, *p* < 0.0001) in the probiotic group compared to the placebo group. Across both groups, as self-reported muscle soreness scores and salivary CRP (C-reactive protein) concentrations increased, sleep quantity, quality and motivation scores decreased. Conversely, as muscle soreness scores and CRP decreased, sleep quantity and quality, and motivation scores improved.
Ching Huang, 2019 Taiwan -double-blind experimental design	Triathletes 18 subjects for Study I 16 subjects for study II Probiotic group (*n* = 9) STUDY I 20.2 ± 0.7 years STUDY II 22.3 ± 1.2 years Placebo group (*n* = 9) STUDY I 21.1± 1.5 years STUDY II 20.1 ± 0.3 years	Lyophilized *L. plantarum* PS128 Dose: twice capsules per day (3 × 10^10^ CFU/day) −4 weeks (STUDY I) −3 weeks (STUDY II)	Body composition evaluated using DEXA (dual-energy X-ray absorptiometer). Anaerobic and aerobic capacities evaluated using a 30-s Wingate anaerobic kinetic Test and VO2 max endurance test. Muscle damage evaluated using Biochemical Indices, such as CK, LDH, protein carbonyl, myioglobin. Muscle fatigue evaluated using Biochemical Indices, such as Ammonia, Lactate and FFA.	*L. plantarum* PS128 supplementation, combined with training, can significantly alleviate oxidative stress (such as creatine kinase, Thioredoxin, and Myeloperoxidase indices) after a triathlon (*p* < 0.05). This effect is possibly regulated by a 6–13% decrease of indicated pro-inflammation (TNF-α, interleukin-6, and interleukin-8) cytokines (*p* < 0.05) and 55% increase in anti-inflammation (interleukin-10) cytokines (*p* < 0.05) after intensive exercise stimulation. In addition, *L. plantarum* PS128 can also substantially increase 24–69% of plasma-branched amino acids (*p* < 0.05) and elevate exercise performance, as compared to the placebo group (*p* < 0.05). There was no significant difference in body composition between the probiotic group and placebo group pre- and post-supplementation (*p* ≤ 0.05).
Ching Huang, 2020 Taiwan -double-blind experimental design	Male Triathletes Probiotic group (*n* = 10) 21.9 ± 1.4 years Placebo group (*n* = 10) 21.6 ± 1.3 years	*L. plantarum* PS128 Dose: a single capsule twice per day, equivalent to 3 × 10^10^ CFU/day −4 weeks	Maximal oxygen consumption and exercise performance evaluated using a treadmill (Pulsar, h/p/cosmos, Germany) and an auto respiratory analyzer K4b2 (Cosmed, Concord, CA, USA). Body composition evaluated using DEXA (dual-energy X-ray absorptiometer).	*L. plantarum* PS128 supplementation was associated with an improvement in endurance running performance through microbiota modulation and related metabolites, but not in maximal oxygen uptake. The probiotic group could significantly elevate endurance performance by the treadmill exercise protocol; the performance could increase by about 130% as compared to the placebo group (*p* = 0.0035). However, at the end of the study, the VO2 max and body composition (bone, fat, and lean percentage) demonstrated no significant difference between groups in the gas and DEXA analysis.
Komano, 2018 Japan -randomized, placebo-controlled, double-blinded trial	Healthy male athletes Probiotic group (*n* = 26) 20.8 ± 0.8 years Placebo group (*n* = 24) 20.5 ± 0.8 years	cells of heat-killed *Lactococcus lactis* strain plasma Dose: a capsule containing 100 billion cells −13 days	Physical condition, fatigue, articular pain, lassitude, and muscle pain evaluated using a daily questionnaire.	CD86 (Cluster of Differentiation 86) as a maturation marker on pDC (plasmacytoid dendritic cells) was significantly increased in the probiotic group. Moreover, the cumulative days of fatigue were significantly fewer in the probiotic group.
Pugh, 2020 UK -randomized, double-blind, placebo-controlled crossover trial	Trained male cyclists (*n* = 7) 23 ± 4 years	Active strains *Lactobacillus acidophilus* (CUL60), *Lactobacillus acidophilus* (CUL21), *Bifidobacterium bifidum* (CUL20), and *Bifidobacterium animalis* subsp. *lactis* (CUL34; Proven Probiotics, Port Talbot, UK) During exercise, subjects consumed a 10% CHO drink enriched with the stable isotope [U-13C] glucose (CK Isotopes, Ibstock, UK). Maltodextrin (176.4 g; Myprotein Inc., Northwich, UK) and 3.6 g [U-13C] glucose Dose: a capsule containing 25 billion CFU-two, 28-day, periods of supplementation, separated by a 14-day washout period.	Exercise trials made using time trial	Probiotics led to minimal increases in absorption and oxidation of the ingested maltodextrin and small reductions in fat oxidation, whereas having no effect on subsequent time-trial performance. During the 100-kJ time trial, there was no significant difference in the time to complete between placebo group (308 ± 69 s) and probiotic group (301± 74 s; *p* = 0.714).
Salleh, 2021 Malaysia -randomized, placebo-controlled study	Badminton Players Probiotic group (*n* = 15) 19.5 ± 1.0 years Placebo group (*n* = 15) 19.9 ± 1.3 years	*Lactobacillus casei Shirota* Dose: drink containing 3 × 10^10^ CFU −6 weeks	Body composition evaluated using the InBody 500 bioelectrical impedance analyser. Aerobic Capacity evaluated using a 20-m multi-stage shuttle run test. Hand strength evaluated using the handgrip test. Leg power assessment measuring the distance of the most extreme point the subject could reach with their arm by jumping. Speed evaluated using a 40-m dash. Agility evaluated using a t-test.	Supplementation of probiotics improved aerobic capacity in probiotic group by 5.9% (*p* < 0.001) but did not influence the speed, strength, leg power and agility.
Sawadaa, 2019 Japan -randomized, double-blind, and placebo-controlled parallel group study	Male university Ekiden (long-distance relay race) runners Probiotic group (*n* = 24) 19.8 ± 1.4 years Placebo group (*n* = 25) 20.1 ± 1.1 years	Heat-inactivated *Lactobacillus gasseri* CP2305 Dose: 200 mL of beverages containing 1 × 10^10^ bacterial cells −12 weeks	Fatigue evaluated using Fatigue Scales	Daily CP2305 intake was effective in recovering from fatigue during the vigorous training period. Furthermore, administration of CP2305 improved the richness and evenness of the gut microbial ecosystem and prevented the stress-induced changes in gene expression of peripheral blood leucocytes. No significant difference in physical performance was found between the probiotic group and placebo group.
Schreiber, 2021 Israel-randomized, double-blind, two-arm, placebo-controlled trial design	Male cyclists, ranked elite or category 1 level competitions Probiotic group (*n* = 11) 25.9 ± 4.6 years Placebo group (*n* = 16) 29.5 ± 6.2 years	(≥) 4.3×10^9^ CFU *Lactobacillus helveticus Lafti* L10 (28.6%), ≥4.3 × 10^9^ CFU *Bifidobacterium animalis* ssp. *lactis Lafti* B94 (28.6%), ≥3.9 × 10^9^ CFU *Enterococcus faecium* R0026 (25.7%), ≥2.1 × 10^9^ CFU *Bifidobacterium longum* R0175 (14.3%) and ≥0.4 × 10^9^ CFU *Bacillus subtilis* R0179 (2.8%) Dose: 15 billion CFU −90 days	Body composition assessed using Skyndex Electronic Skinfold Caliper (Caldwell, Justiss & Co., Inc., Fayetteville, AR, USA), measuring 4 skinfolds (triceps, biceps, subscapularis, iliac crest) in triplicates and the average of each skin fold was used to calculate body density and percent body fat was calculated using the Siri equation. VO2max measured using Metalyzer 3B (Cortex Biophysik GmbH, Leipzig, Germany) metabolic cart and determined following personalized graded exercise protocol. Fatigue evaluated using Time to fatigue (TTF) test	Mean rate of perceived exertion (RPE) values during the TTF (test and time-to-fatigue) were lower in the probiotic group (ΔRPE: −0.3 ± 0.9 vs. 0.8 ± 1.5, *p* = 0.04). No significant changes were measured between and within groups in VO_2_max and TTF values, mean levels of C-reactive protein, interleukin-6 and tumour necrosis factor-alpha values following treatment.
Shing, 2014 Australia -double-blind, cross-over trial	Male runners Probiotic group (*n* = 5) Placebo group (*n* = 5) 27 ± 2 years	7.4 billion CFU of *Lactobacillus acidophilus*, 15.55 billion CFU of *L. rhamnosus*, 9.45 billion CFU of L. casei, 3.15 billion CFU of *L. plantarum*, 1.35 billion CFU of *L. fermentum*, 4.05 billion CFU of *Bifidobacterium lactis*, 1.35 billion CFU of *B. breve*, 0.45 billion CFU of *B. bifidum* and 2.25 billion CFU of *Streptococcus thermophilus* Dose: 1 capsule/day (45 billion CFU) −4 weeks	VO_2_ max evaluated using a test involving an incremental treadmill run to fatigue starting at 10 km h^−1^, 0% gradient with the speed increasing by 1 km h^−1^ each minute until a speed of 18 km h^−1^. After 1 min at 18 km h^−1^, the treadmill gradient was increased by 1% each minute until volitional fatigue. Fatigue evaluated time-to-fatigue run test	Probiotics supplementation significantly increased run time to fatigue (min:s 37:44 ± 2:42 versus 33:00 ± 2:27; *p* = 0.03, d (Cohen’s effect size) = 0.54).
Smarkusz-Zarzecka, 2020 Poland -randomised, double-blind study	Long-Distance Runners Probiotic group (*n* female = 14; 37.21 ± 8.09 years); (*n* male = 20; 40.85 ± 8.32 years) Placebo group (*n* female = 6; 33.33 ± 8.73 years) (*n* male = 26; 38.61 ± 8.84 years)	*Bifidobacterium lactis* W52, *Lactobacillus brevis* W63, *Lactobacillus casei* W56, *Lactococcus lactis* W19, *Lactococcus lactis* W58, *Lactobacillus acidophilus* W37, *Bifidobacterium bifidum* W23 and *Lactobacillus salivarius* W24 Dose: 2 capsules of the supplement twice a day- A capsule contains 2.5 × 10^9^ CFU/g −3 months	Body Composition evaluated using the InBody770 analyser. Cardiorespiratory Fitness evaluated using the Fitmate MED device and a medical treadmill adapted. The Bruce protocol treadmill test was used.	A statistically significant increase in maximum oxygen uptake VO_2_max (*p* = 0.017), minute ventilation (Ve) (*p* = 0.013), functional capacity (FC) (*p* = 0.036), breathing reserve (*p* = 0.020) and exercise capacity (*p* = 0.036) was observed in the group of men taking the probiotic supplement. In the group of women taking the probiotic supplement, a decrease in body fat (in kilograms and percentages) and visceral fat (VAT) was observed, but the differences were not statistically significant.
Strasser, 2016 Austria -randomized, double-Blinded, placebo-controlled Trial	Trained athletes Probiotic group (*n* = 14) 25.7 ± 3.5 years Placebo group (*n* = 15) 26.6 ± 3.5 years	*Bifidobacterium bifidum* W23, *Bifidobacterium lactis* W51, *Enterococcus faecium* W54, *Lactobacillus acidophilus* W22, *Lactobacillus brevis* W63, and *Lactococcus lactis* W58 (Ecologic^®^ Performance, Winclove B.V., Amsterdam, The Netherlands). The total cell count was adjusted to 2.5 × 10^9^ colony-forming units (CFU) per gram Dose: 1 sachet of 4 g per day, which is equivalent to 1 × 10^10^ CFU/day −3 months	Athletic performance evaluated using n incremental cycle ergometer exercise test until Exhaustion. Body composition evaluated using the bioelectrical impedance analysis (BIA) method (BIA-2000-M, Data Input, Pöcking, Germany).	Data indicate reduced exercise-induced tryptophan degradation rates in the probiotic group. Daily supplementation with probiotics, however, did not benefit athletic performance and body composition. Analysis of training loads indicated that the weekly training of the aerobic system, mainly continuous endurance training at moderate intensity (60% to 80% VO_2_max), varied significantly between the group: the means were significantly higher in the probiotics group as compared to the placebo (8.0 ± 2.3 and 6.6 ± 4.3 h per week endurance training, respectively).
Toohey, 2018 Tennessee -double-blind, placebo-controlled, randomized study	Female athletes (volleyball, soccer) Probiotic group (*n* = 11) Placebo group (*n* = 12) 19.6 ± 1.0 years	DE111 (genome sequenced and clinically tested strain of *Bacillus subtilis*) Dose: 5 billion CFU/day −10 weeks	Body Composition evaluated using air displacement plethysmography using the BODPOD (COSMED, Rome, Italy), multifrequency bioelectrical impedance analysis (BIA) using the InBody 570 Body Composition Analyzer device (Biospace, Inc., Seoul, Republic of Korea), the 3-compartment water (3C-W) model described by Siri (32). Performance evaluated using: dynamic strength test, isometric strength test, vertical jump test, and pro-agility test.	Significant (*p* ≤ 0.05) main effects for time were observed for improved squat 1RM (1 repetition maximum), deadlift 1RM, bench press 1RM, vertical jump, RF MT (rectus femoris muscle thickness), and BF% (body fat%). Of these, a significant group 3-time interaction was noted for BF% (*p* = 0.015), where greater reductions were observed in probiotic group (−2.05 ± 1.38%) compared with placebo group (−0.2 ± 1.6%).
Townsend, 2018 USA -double-blind, placebo-controlled, randomized study	Male baseball athletes Probiotic group *n* = 13 Placebo group *n* = 12 20.1 ± 1.5 years	*Bacillis subtilis* DE111 (Deerland Enzymes, Kennesaw, GA, USA) Dose: a capsule containing 1.2 billion CFU −12 weeks	Body Composition evaluated using air displacement plethysmography using the BODPOD (COSMED, Rome, Italy), multifrequency bioelectrical impedance analysis (BIA) using the InBody 570 Body Composition Analyzer device (Biospace, Inc., Seoul, Republic of Korea), the 3-compartment water (3C-W) model described by Siri (32). Performance evaluated using: dynamic strength test, Ten-Yard Sprint, Pro-Agility Test, Standing Long Jump.	There were no group differences observed between the probiotic group and placebo group for any measure of strength, performance or body composition. Collectively, significant improvements (*p* < 0.001) were observed in squat 1RM, deadlift 1RM, pro-agility, and standing long jump as a result of 12 weeks of offseason training, while no improvement (*p* = 0.312) in 10-yard sprint time was found.

**Table 3:** Methodological quality of the studies using the tool RoB 2.0.

First Author Name	Randomization Process	Deviation from the Intended Intervention	Missing Results Data	The Measurement Result	Selection of the Result Reported	General Trend
Harnett, 2020 [33]	Low	Low	Low	Low	Low	Low
Ching Huang, 2019 [34]	Low	Low	Some concerns	Low	Low	Some concerns
Ching Huang, 2020 [35]	Low	Low	Some concerns	Low	Low	Some concerns
Komano, 2018 [36]	Low	Low	Low	High	Low	High
Pugh, 2020 [37]	Low	Low	Some concerns	Low	Low	Some concerns
Salleh, 2021 [38]	Low	Low	Low	Low	Low	Low
Sawadaa, 2019 [39]	Low	Low	Low		Low	Low
Schreiber, 2021 [40]	Some concerns	Low	Some concerns	Low	Low	High
Shing, 2014 [41]	Low	Low	Low	Low	Low	Low
Smarkusz-Zarzecka, 2020 [42]	Low	Low	Low	Low		Low
Strasser, 2016 [43]	Low	Low	Low	Low	Low	Low
Toohey, 2018 [44]	Low	Low	Low	Low	Low	Low
Townsend, 2018 [45]	Low	Low	Low	Low	Low	Low

## Data Availability

Not applicable.
